# Adrenergic Modulation of Erythropoiesis After Trauma

**DOI:** 10.3389/fphys.2022.859103

**Published:** 2022-03-18

**Authors:** Jennifer A. Munley, Lauren S. Kelly, Alicia M. Mohr

**Affiliations:** Sepsis and Critical Illness Research Center, Department of Surgery, University of Florida, Gainesville, FL, United States

**Keywords:** erythropoiesis, trauma, catecholamine, bone marrow, beta blockade, alpha agonist

## Abstract

Severe traumatic injury results in a cascade of systemic changes which negatively affect normal erythropoiesis. Immediately after injury, acute blood loss leads to anemia, however, patients can remain anemic for as long as 6 months after injury. Research on the underlying mechanisms of such alterations of erythropoiesis after trauma has focused on the prolonged hypercatecholaminemia seen after trauma. Supraphysiologic elevation of catecholamines leads to an inhibitive effect on erythropoiesis. There is evidence to show that alleviation of the neuroendocrine stress response following trauma reduces these inhibitory effects. Both beta blockade and alpha-2 adrenergic receptor stimulation have demonstrated increased growth of hematopoietic progenitor cells as well as increased pro-erythropoietic cytokines after trauma. This review will describe prior research on the neuroendocrine stress response after trauma and its consequences on erythropoiesis, which offer insight into underlying mechanisms of prolonged anemia postinjury. We will then discuss the beneficial effects of adrenergic modulation to improve erythropoiesis following injury and propose future directions for the field.

## 1 Introduction

Trauma patients develop persistent anemia after insult despite blood transfusions as a result of impaired erythropoiesis (Robinson et al., 2006). This anemia and subsequent need for blood transfusion is associated with greater mortality independent of the severity of shock (Malone et al., 2003). The etiology underlying this persistent anemia after trauma appears to be independent of plasma erythropoietin, given levels of plasma erythropoietin two to eight times higher than healthy controls after injury with inappropriately low plasma reticulocyte levels (Livingston et al., 2003). Phenomena associated with this post-injury anemia include systemic inflammation, mobilization of hematopoietic progenitor cells (HPC) which re-locate to sites of tissue injury, decreased bone marrow cellularity and erythroid progenitor growth suppression, and altered iron metabolism. The underlying mechanisms behind each of these phenomena continue to be investigated. Both beta blockade and alpha-2 adrenergic receptor stimulation have demonstrated increased growth of hematopoietic progenitor cells as well as increased pro-erythropoietic cytokines after trauma. This review will provide an overview of the current body of research that exists surrounding the underlying mechanisms of erythropoietic suppression after injury; in particular, the role of persistent hypercatecholaminemia and the available evidence to support adrenergic modulation in alleviating the neuroendocrine stress response.

## 2 Mediators of Persistent Anemia After Trauma

### 2.1 Systemic Inflammation and Red Blood Cell Damage

Traumatic injury induces a state of systemic inflammation associated with release of proinflammatory cytokines (Dinarello, 2000). Trauma patients exhibit elevations in circulating pro-inflammatory cytokines like C-reactive protein (CRP), interleukin-1α and 1β (IL-1α and IL-1β), interleukin-6 (IL-6), nitric oxide (NO), and tumor necrosis factor alpha (TNFα) immediately after injury (Volpin et al., 2014). This occurs in conjunction with decreased levels of anti-inflammatory cytokines such as interleukin-10 (IL-10), which stimulates erythroid colony growth (Wang et al., 1996). Proinflammatory signaling factors promote myeloid lineage expansion at the expense of erythroid progenitors (IL-1α, IL-1β), induce production of iron-sequestering peptide hepcidin (IL-6) that leads to inappropriate hypoferremia in the setting of acute blood loss anemia, and directly inhibit erythroid progenitor growth (TNFα) (Johnson et al., 1991; Rusten and Jacobsen, 1995; Nemeth et al., 2004; Raj, 2009; Pietras et al., 2016). Nitric oxide signaling results in increased IL-6 and HPC pro-mobilization factor, in addition to granulocyte-colony stimulating factor (G-CSF) (Hierholzer et al., 1998). Elevated CRP is an acute phase reactant and indicator of inflammation which is associated with anemia (Heidari et al., 2015). The alteration of these cytokine levels after trauma negatively impacts erythropoiesis in these patients.

Traumatic injury also causes damage to red blood cells. A study of trauma and hemorrhagic shock in rats demonstrated that red blood cells have decreased deformability, measured by elongation index, after shock which correlates with the degree of physiologic insult (Zaets et al., 2003). Another rat study of trauma and hemorrhagic shock showed that red blood cell damage also contributes to organ hypoperfusion leading to organ failure, possibly as a result of erythrophagocytosis in tissues, a phenomena observed in septic subjects (Machiedo et al., 2009). Finally, red blood cell metabolism is altered after hemorrhagic shock, with a dependence on glutamine for glutathione synthesis and alanine transamination (Reisz et al., 2017). These findings illustrate how trauma changes red blood cells and impacts organ perfusion independent of the hypovolemic state of hemorrhagic shock.

### 2.2 Mobilization of Hematopoietic Progenitor Cells and Impacts on Bone Marrow Cellularity and Erythroid Progenitor Growth

**FIGURE 1:**
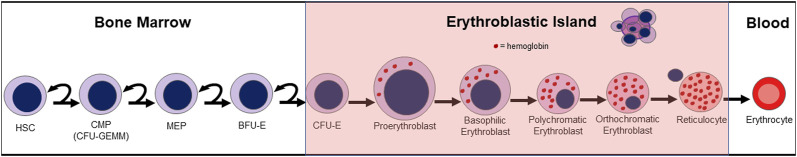
Normal erythropoiesis. HSC (hematopoietic stem cell); CMP (common myeloid progenitor); CFU-GEMM (multipotential colony-forming unit); MEP (megakaryocyte erythroid progenitor); BFU-E (blast-forming unit-erythroid); CFU-E (colony-forming unit-erythroid).

Trauma induces mobilization of HPCs from the bone marrow to the peripheral blood and sites of tissue injury (Livingston et al., 2003; Badami et al., 2007; Shah et al., 2009; Cook et al., 2013). In animal studies, HPC mobilization to injured tissues is essential for accelerated wound healing (Badami et al., 2007; Shah et al., 2009; Hannoush et al., 2011). Plasma from trauma patients demonstrate sustained peripheral blood HPC for up to 10 days after injury (Cook et al., 2013). In normal erythropoiesis, multipotent progenitor cells differentiate into multipotential colony-forming units (CFU-GEMM), erythroid burst-forming units (BFU-E), and erythroid colony-forming units (CFU-E), and then erythroblasts, ultimately developing into erythrocytes (Figure 1). Erythroid progenitors such as these are shown to be consistently suppressed in both trauma patients and animal studies (Livingston et al., 2003; Badami et al., 2007; Loftus et al., 2018b). In contrast, other proteins like stem cell factor (SCF) and B-cell lymphoma-extra-large (Bcl-xL) have been linked to the promotion of erythropoiesis (Alter et al., 1992; Afreen et al., 2020). The damage-associated high-mobility group box-1 (HMGB1) protein suppresses bone marrow cellularity and mediates an anemia that has been reversed with administration of anti-HMGB1 monoclonal antibodies in mice (Valdes-Ferrer et al., 2016). Suppression of CFU-E has also been observed when human bone marrow was cultured with HMGB1 *in vitro* (Dulmovits et al., 2022).

A variety of factors affect the balance between retention and mobilization of hematopoietic progenitor cells in the bone marrow. Granulocyte colony-stimulating factor (G-CSF) is well established to promote hematopoietic progenitor cell mobilization (Tesio et al., 2011). It induces stromal-cell derived factor-1 (SDF-1) secretion into the circulation to attract HPC (Saba et al., 2015). Additional factors which promote HPC mobilization include hepatocyte growth factor (HGF), tyrosine-protein kinase Met (c-Met), matrix-metalloproteinase-9 (MMP-9), and matrix-metalloproteinase-2 (MMP-2) (Heissig et al., 2002; Tesio et al., 2011; Klein et al., 2015; Tari et al., 2017). Tissue inhibitor of metalloproteinase-1 (TIMP-1) and tissue inhibitor of metalloproteinase-2 (TIMP-2) are metalloproteinase inhibitors; TIMP-1 binds MMP-9 and TIMP-2 binds MMP-2 and subsequently inhibit their actions such as HPC mobilization (Parks et al., 2004). In murine studies, plasma corticosterone, a glucocorticoid, promotes HPC migration through glucocorticoid receptor Nr3c1-dependent signaling (Pierce et al., 2017). Murine studies also demonstrate that high mobility group box-1 (HMGB1) mediates macrophage secretion of G-CSF, inducing HPC mobilization (Xiang et al., 2012). Stromal cell-derived factor-1 (SDF-1), also known as C-X-C motif chemokine ligand 12 (CXCL12), and its receptor C-X-C chemokine receptor type 4 (CXCR4) which is present on hematopoietic progenitor cells, mediate retention of HPC in bone marrow stroma (Greenbaum and Link, 2011; Bonig and Papayannopoulou, 2012). In addition, HPC express very late antigen-4 (VLA-4), an adhesion molecule which binds to vascular cell adhesion molecule-1 (VCAM-1) to retain HPC in bone marrow (Bonig and Papayannopoulou, 2012). Neutrophil elastase cleaves VCAM-1 to induce HPC mobilization (Levesque et al., 2001). These factors affecting HPC mobilization from the bone marrow play a pivotal role in understanding the underlying mechanisms of bone marrow dysfunction after trauma, manifesting as persistent anemia.

### 2.3 Hypercatecholaminemia After Trauma

Norepinephrine and epinephrine are catecholamines secreted by the adrenal medulla and act on adrenergic receptors (sometimes referred to as adrenoreceptors), which are classified into α_1_, α_2_, β_1_, β_2_, and β_3_ subtypes (Maestroni, 2020; Wu et al., 2021). Norepinephrine has a greater affinity for alpha receptors and epinephrine has a stronger affinity for beta receptors (Maestroni, 2020). Activation of α_1_ receptors result in peripheral vasoconstriction, whereas centrally located α_2_ receptor activation inhibits the release of additional norepinephrine (Wu et al., 2021). Beta-1 receptors are the predominant adrenergic receptor in the heart and activation results in increased rate of nodal firing and conduction as well as increased cardiac contractility (Wu et al., 2021). Beta-2 receptors are found at a lower density compared to beta-1 receptors in the heart; they are also located in smooth muscle, including smooth muscle of the airways where they play a clinical role in bronchoconstriction when activated (Wu et al., 2021). Beta-3 receptors are involved in lipolysis in adipose tissue; they are also present in the myocardium but have controversial effects on cardiac function (Arioglu-Inan et al., 2019; Schena and Caplan, 2019). Beta-3 receptor activation in human studies have either demonstrated minimal cardiac changes or negative inotropic effects (Arioglu-Inan et al., 2019). Hypercatecholamine states result in activation of these receptors and induction of downstream effects, including increased cardiac contractility and smooth muscle constriction.

In humans and animals, elevated catecholamine levels persist after injury (Fonseca et al., 2004; Loftus et al., 2018b). Rodent trauma models demonstrate significantly increased urine norepinephrine which persists 7 days after injury (Bible et al., 2015a; Alamo et al., 2017b; Loftus et al., 2018a). Trauma patients admitted to the surgical intensive care unit (SICU) demonstrate significantly increased urine norepinephrine for 10 days and epinephrine for 7 days compared to healthy controls (Fonseca et al., 2004). An observational study of blunt trauma patients also demonstrated significantly higher circulating norepinephrine compared to control patients who underwent total hip replacement (Loftus et al., 2018b). Similar to trauma, burn injury has been shown to lead to significant elevation of cortisol, catecholamines, cytokines and acute phase proteins for up to 3 years post-burn (Jeschke et al., 2011). This state of hypercatecholaminemia has varying physiologic implications, including increased expression of erythropoietic-inhibiting cytokines such as IL-1α, interleukin-2 (IL-2), IL-6, IL-10 and transforming growth factor beta (TGF-β) (Saba et al., 2017). Given this rise in catecholamine levels and its effects on cytokine release, hypercatecholaminemia has been a point of interest for further investigation as a contributing factor to persistent anemia after trauma.

### 2.4 Iron Metabolism

Iron is an essential component in erythropoiesis (Weiss et al., 2019). Every second, almost two million erythrocytes are produced in the bone marrow, which requires up to 30 mg of iron daily for synthesis of hemoglobin (Gkouvatsos et al., 2012; Weiss et al., 2019). Erythropoiesis becomes iron dependent during hemoglobin synthesis, which begins at the proerythoblast stage (Weiss et al., 2019). As a result, erythropoiesis can be substantially impacted by states of functional iron deficiency.

**FIGURE 2:**
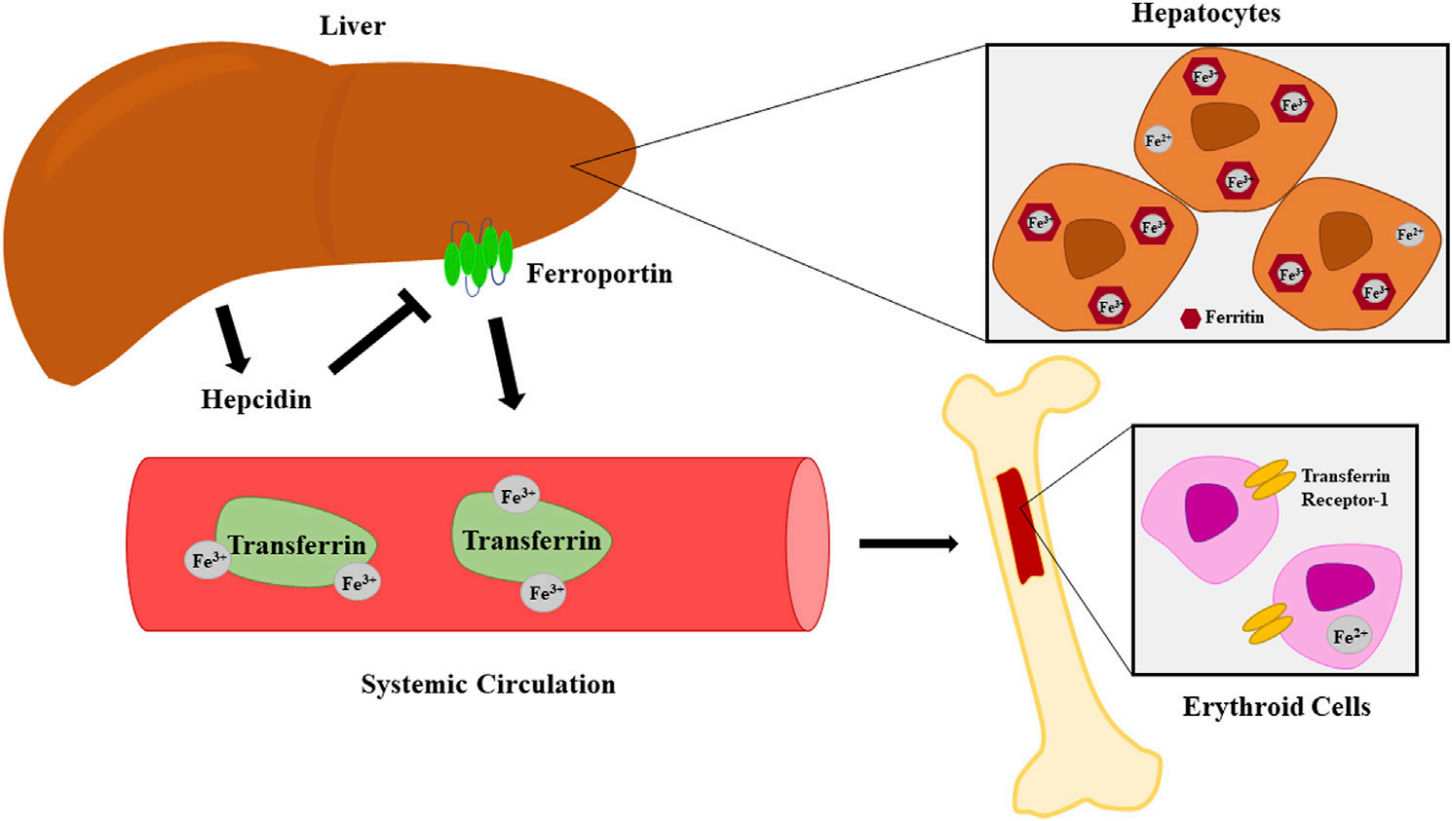
Iron metabolism and transport from hepatocytes to erythroid cells. Hepcidin induces internalization and degradation of the iron-transport protein ferroportin.

Inflammatory states disrupt the iron homeostasis necessary for adequate erythropoiesis (Sangkhae and Nemeth, 2017). The liver plays a key role in these processes. It is the predominant producer of hepcidin, a peptide hormone that serves as a gatekeeper for release of free iron by causing internalization and degradation of iron-transport protein ferroportin (Figure 2) (Sangkhae and Nemeth, 2017; Lanser et al., 2021). It is also a major source of IL-6, which increases hepcidin expression and in turn promotes iron sequestration (Sangkhae and Nemeth, 2017). Additionally, IL-1β is another potent inducer of hepcidin expression (Gkouvatsos et al., 2012). The widespread inflammation and increase in systemic hepcidin seen in trauma patients lead to iron sequestration and reduced availability of the raw materials necessary for hemoglobin synthesis during erythropoiesis (Sangkhae and Nemeth, 2017; Loftus et al., 2018b).

### 2.5 Hypercatecholaminemia and Erythropoiesis

*In vitro* studies demonstrate the direct effects of hypercatecholaminemia on erythropoiesis. One study which cultured iliac crest bone marrow from trauma patients demonstrated BFU-E and CFU-E growth decline at supraphysiologic catecholamine levels, starting at 10^−7^ M (Fonseca et al., 2004). This is similar to levels of circulating norepinephrine measured in severely injured trauma patients with a mean of 44.1 ng/ml (2.6 × 10^−7^ M) (Apple et al., 2020a). However, this decrease in erythroid progenitor cell growth was not observed in bone marrow depleted of stroma, suggesting that bone marrow stroma plays an essential role in the maturation of erythroid progenitor cells (Fonseca et al., 2004). A follow-up study of human bone marrow cells cultured in the presence of epinephrine or norepinephrine at increasing doses demonstrated a decline in BFU-E and CFU-E in a dose-dependent manner at supraphysiologic levels (Fonseca et al., 2005). It is known that the bone marrow is innervated with sympathetic fibers which affect erythropoiesis (Maestroni, 2020). When chemical sympathectomy was performed in rodents and norepinephrine pumps implanted for administration of increasing doses of norepinephrine for 5 days, rodents had decreased erythroid progenitor growth in a dose-dependent fashion when bone marrow was cultured *in vitro* (Penn et al., 2011). These *in vitro* studies suggest dose-dependent effects of norepinephrine and epinephrine on erythroid progenitor growth and erythropoiesis.

Effects of catecholamines on terminal erythropoiesis continue to be investigated. Terminal erythropoiesis is the process by which erythroblasts accumulate hemoglobin, mature, and finally expel their nuclei to become reticulocytes, as shown in Figure 1. This process occurs on the erythroblastic island (EBI), which is comprised of a central macrophage surrounded by developing CFU-E, erythroblasts, and recently enucleated reticulocytes (Chasis and Mohandas, 2008). These islands can be found within both bone marrow and spleen, where they serve as a niche for the proliferation, differentiation and enucleation of erythroid precursors ultimately leading to the production of erythrocytes (Chasis and Mohandas, 2008). Bone marrow-derived rabbit erythroid cells undergo an uncoupling of their beta adrenergic receptors when cultured in catecholamine-enriched media; this uncoupling occurs early in erythroblast development (Setchenska and Arnstein, 1983; Setchenska et al., 1983). Much remains unknown about what effects adrenergic modulation could exhibit on terminal erythropoiesis and EBIs.

Some mechanisms behind these effects of supraphysiologic levels of catecholamines on erythropoiesis have been identified. Sympathetic nervous system stimulation is associated with release of G-CSF which in turn promotes HPC mobilization (Katayama et al., 2006; Saba et al., 2015; Gao et al., 2021). Particularly, β-2 and β-3 adrenoreceptors are critical in HPC mobilization, likely associated with inhibition of SDF-1 (Mendez-Ferrer et al., 2010). In addition, it has been shown that HPC express α-1, α-2, and β-2 adrenergic receptors, of which there is higher expression of β-2 on HPC when mobilized via G-CSF, suggesting that mobilization may increase catecholamine sensitivity (Muthu et al., 2007; Spiegel et al., 2007).

The use of adrenergic modulation in the trauma population has established clinical relevance in the setting of hypercatecholaminemia. Elevated levels of epinephrine are associated with increased mortality after trauma (Johansson et al., 2012). The use of beta-adrenergic blockade in trauma patients improves mortality, possibly by decreasing hypermetabolism and cerebral oxygen requirement (Arbabi et al., 2007). Beta blockade in trauma patients has also decreases IL-6 levels (Friese et al., 2008). Burn patients treated with beta antagonists demonstrate improved wound healing and a decreased surface area required for skin grafting (Mohammadi et al., 2009; Ali et al., 2015). In addition, burn patients who are treated with beta blockade have attenuated hypermetabolism and reversal of muscle-protein catabolism (Herndon et al., 2001). With adrenergic modulation already in use in this patient population, and given the effects of hypercatecholaminemia on erythropoiesis, the application of alpha agonism and beta blockade as therapeutic interventions to prevent persistent anemia after injury were of particular interest. A thorough literature search of studies involving adrenergic modulation of erythropoiesis after trauma was performed to identify articles for this comprehensive review. While many of these studies originate from the authors, all studies that were relevant to this topic have been included.

## 3 Adrenergic Modulation

### 3.1 Alpha Agonism Following Trauma

Alpha agonist medications act by inhibiting sympathetic outflow from the central nervous system, which has been shown to decrease both cerebrospinal and peripheral norepinephrine and normetanephrine and reduce plasma epinephrine (Sullivan et al., 1986). Clonidine is a widely utilized medication that stimulates α-2 adrenoceptors in the central nervous system to inhibit sympathetic outflow, resulting in reductions in heart rate, blood pressure, and peripheral vascular resistance (Campese et al., 1980; Sullivan et al., 1986; Dorman et al., 1997). In general, patients who receive clonidine (not limited to trauma patients) exhibit reduced circulating plasma norepinephrine and epinephrine (Campese et al., 1980; Sullivan et al., 1986; Payen et al., 1990; Dorman et al., 1997). Clonidine is typically used to treat hypertension, but its efficacy in reducing the sympathetic response has made it a drug of interest among researchers seeking novel treatments for persistent anemia in injured patients.

Much of what is known about the effects of alpha agonism on erythropoiesis is based on preclinical studies, particularly on rat models of trauma. This involves either lung contusion (LC), lung contusion with chronic daily restraint stress (LC + CS), hemorrhagic shock (HS), lung contusion with hemorrhagic shock (LCHS), or lung contusion with hemorrhagic shock and chronic daily restraint stress (LCHS + CS). Chronic daily restraint stress alone (CS) is used to identify how stress, meant to simulate SICU stay, affects outcomes. Rodent trauma models represent different injuries, such as blunt trauma (lung contusion), hemorrhage and subsequent stay in a SICU (chronic daily restraint stress). Despite these parallels in the model to human blunt trauma and hospitalization, patients often have multiple injuries including fractures which are not represented by this model such as fractures and traumatic brain injury among others. Rat models also do not involve post-operative or post-injury infection, such as wound infection or pneumonia. In addition, rats who undergo hemorrhagic shock are resuscitated by re-transfusion of their own blood, different from human resuscitation with allogenic blood. Another consideration is that hemorrhagic shock in rats does not produce the same tachycardia observed in humans in hemorrhagic shock (Weber et al., 2019). A summary of animal studies which are reviewed can be found in Table 1. These models have been validated to effectively influencing a variety of factors involved in erythropoiesis as will be discussed in the following sections. The models of LC, LCHS and LCHS + CS affect expression of hematopoietic cytokines, HMGB1, SCF, Bcl-xL; urine norepinephrine; hemoglobin; plasma G-CSF; HPC mobilization; plasma corticosterone; bone marrow cellularity; and erythroid progenitor cell growth (Alamo et al., 2017b; Loftus et al., 2019; Loftus et al., 2020).

**TABLE 1:** Major studies of adrenergic modulation of erythropoiesis in rodents (rats or mice) who were subject trauma. Major effects demonstrate significant changes (increased or decreased) represented by arrows compared to untreated counterparts.

References	Trauma Model(s)	Length of model	Medication(s)	Administration	Duration of treatment	Major effects
Alamo et al. (2017a)	LCHS^†^	7 days	Propranolol	10 mg/kg daily intraperitoneal	7 days	↑ bone marrow cellularity^†^^‡^
	LCHS + CS^‡^					↑ erythroid progenitor growth^†^^‡^
						↓ plasma erythropoietin^†^^‡^
						↑ plasma hepcidin^†^^‡^
						↑ liver ferroportin^†^^‡^
						↑ bone marrow transferrin^†^^‡^
						↑ bone marrow TFR-1^†^^‡^
						↑ hemoglobin^†^^‡^
Alamo et al. (2017b)	CS^§^	7 days	Clonidine	75 μg/kg daily intraperitoneal	7 days	↓ urine norepinephrine^§^^‡^
	LCHS^†^					↑ hemoglobin^†^^‡^
	LCHS +CS^‡^					↓ plasma G-CSF^§^^†^^‡^
						↓ HPC mobilization^§^^‡^
						↑ bone marrow cellularity^§^^†^^‡^
						↑ erythroid progenitor growth^§^^†^^‡^
Apple et al. (2020b)	LCHS + CS^‡^	7 days	Propranolol	10 mg/kg daily intraperitoneal	7 days	↑ Rno-miR-27a and mR-25^‡^
						↓ bone marrow IL-1β^‡^
						↓ bone marrow TNF-α^‡^
						↓ bone marrow NO^‡^
						↓ plasma CRP^‡^
Baranski et al. (2011)	LC*	3 h	Propranolol	10 mg/kg daily intraperitoneal	Once	↑ bone marrow cellularity* ^# †^ (3 h, 24 h)
	HS^#^	24 h				↓ HPC mobilization^# †^ (3h, 24 h)
	LCHS^†^					↓ plasma G-CSF^# †^ (3 h, 24 h)
						↓ plasma MMP-9^†^ (3 h)
Baranski et al. (2013)	LCHS^†^	3 h	Propranolol	1, 2.5, 5, or 10 mg/kg daily intraperitoneal	Once	↑ bone marrow cellularity^†^ (5, 10 mg/kg; 3h, 7 d)
		7 days			7 days	↑ erythroid progenitor growth^†^ (5, 10 mg/kg; 3h, 7 d)
						↓ HPC mobilization^†^ (5, 10 mg/kg; 3 h)
Beiermeister et al. (2010)	LC*	3 h	Atenolol (β_1_)	5 mg/kg (β_1_, β_2_, β_3_)	3 days prior to injury	↑ bone marrow cellularity* (β_2_, β_3_, β_ns_)
			Butoxamine (β_2_)	10 mg/kg (β_ns_) daily intraperitoneal		↓ HPC mobilization* (β_2_, β_3_, β_ns_)
			SR59230A (β_3_)			
			Propranolol (β_ns_)			
Bible et al. (2015b)	LC*	7 days	Propranolol	10 mg/kg daily intraperitoneal	7 days	↓ HPC mobilization^Δ^^‡^
	LC + CS^Δ^					↓ plasma G-CSF^Δ^^‡^
	LCHS^†^					
	LCHS + CS^‡^					
Doobay et al. (2021)	LC*	7 days	Propranolol	10 mg/kg daily intraperitoneal	7 days	↓ bone marrow HMGB1^‡^
	LCHS^†^					↓ bone marrow G-CSF^‡^
	LCHS + CS^‡^					↓ bone marrow MMP-2^†^^‡^
						↓ bone marrow MMP-9* ^†^^‡^
						↓ plasma MMP-9^‡^
						↓ bone marrow neutrophil elastase ^‡^
						↓ plasma neutrophil elastase* ^†^^‡^
						↓ bone marrow SDF-1*
						↓ bone marrow CXCR4^‡^
						↓ bone marrow VLA-4^‡^
						↓ bone marrow TIMP-1* ^‡^
						↓ bone marrow TIMP-2^†^^‡^
Elhassan et al. (2011)	HS^#^	3 h	Propranolol	10 mg/kg daily intraperitoneal	3 days prior to injury (pre)	↑ erythroid progenitor growth^#^ (pre, post)
					Once (post)	
Hasan et al. (2017a)	Scald burn	7 days	Propranolol	1.2 mg daily subcutaneous	7 days	↓ MafB expression (7d, 14d)
		14 days			14 days	
Hasan et al. (2017b)	Scald burn	7 days	Nadolol (β_1_)	625 µg daily subcutaneous (β_1_)	7 days	↑ hemoglobin (β_2_, β_3_)
			Butoxamine (β_2_)	125 µg daily subcutaneous (β_2_, β_3_)		↑ bone marrow erythroid cells (β_ns_)
			SR59230A (β_3_)	1.2 mg daily subcutaneous (β_ns_)		↓ MafB expression (β_1_,β_2_, β_n_)
			Propranolol (β_ns_)			↑ reticulocytes (β_2_, β_3_)
Loftus et al. (2020)	LC*	7 days	Propranolol (β_ns_)	10 mg/kg daily intraperitoneal (β_ns_)	7 days	↑ hemoglobin^‡^ (α, β_ns_)
	LCHS^†^		Clonidine (α)	75 μg/kg daily intraperitoneal (α)		↓ HPC mobilization^‡^ (α, β_ns_)
	LCHS + CS^‡^					↓ plasma corticosterone^‡^ (α, β_ns_)
						↓ bone marrow MMP-9^‡^ (α, β_ns_) * (α)
						↓ bone marrow c-Met* (α)
Loftus et al. (2019)	LCHS^†^	7 days	Propranolol (β_ns_)	10 mg/kg daily intraperitoneal (β_ns_)	7 days	↓ bone marrow HMGB1^†^^‡^ (α, β_ns_)
	LCHS + CS^‡^		Clonidine (α)	75 μg/kg daily intraperitoneal (α)		↑ bone marrow interleukin-1α^†^^‡^ (α, β_ns_)
						↑ bone marrow interleukin-1β^†^^‡^ (α, β_ns_)
						↑ bone marrow SCF^†^^‡^ (α, β_ns_)
						↑ bone marrow Bcl-xL^†^ (β_ns_) ^‡^ (α, β_ns_)
Miller et al. (2020)	LCHS + CS^‡^	7 days	Atenolol (β_1_)	10 mg/kg daily intraperitoneal (β_1_, β_2_, β_3_)	7 days	↑ hemoglobin^‡^ (β_2_, β_3_)
			Butoxamine (β_2_)			↑ erythroid progenitor growth^‡^ (β_2_, β_3_)
			SR59230A (β_3_)			↓ HPC mobilization^‡^ (β_2_)
						↓ plasma G-CSF^‡^ (β_1_, β_2_, β_3_)
Mohr et al. (2011)	LCHS^†^	24 h	Propranolol	10 mg/kg daily intraperitoneal	Once	↑ erythroid progenitor growth^†^ (24 h, 7d)
		7 days			7 days	↑ hemoglobin^†^ (7d)
Pasupuleti et al. (2014)	LCHS^†^	3 h	Atenolol (β_1_)	10 mg/kg daily intraperitoneal (β_1_, β_2_)	Once	↑ bone marrow cellularity^†^ (β_2_, β_3_; 3 h, 7d)
		7 days	Butoxamine (β_2_)	5 mg/kg daily intraperitoneal (β_3_)	7 days	↑ erythroid progenitor growth^†^ (β_2_, β_3_; 3 h)
			SR59230A (β_3_)			↓ HPC mobilization^†^ (β_2_, β_3_; 3 h)
						↓ plasma G-CSF^†^ (β_3_; 3 h)
Pasupuleti et al. (2012)	LCHS^†^	3 h	Atenolol (β_1_)	5 mg/kg daily intraperitoneal (β_1_, β_2_, β_3_)	Once	↑ bone marrow cellularity^†^ (β_2_, β_3_; 3h, 7 d)
		7 days	Butoxamine (β_2_)		7 days	↑ erythroid progenitor growth^†^ (β_2_, β_3_; 3 h, 7d)
			SR59230A (β_3_)			↑ hemoglobin^†^ (β_3_; 7d)

Agents causing these effects are denoted by α, β_1_, β_2_, β_3,_ β_ns_. Length of the model is defined as time after injury to sacrifice. Duration of treatment with medication indicates post-injury treatment time unless otherwise specified. LC*—lung contusion; HS^#^—hemorrhagic shock; LCHS^†^—lung contusion and hemorrhagic shock; CS^§^—daily chronic stress; LCHS + CS^‡^—lung contusion with hemorrhagic shock and daily chronic stress; and LC + CS^Δ^—lung contusion with daily chronic stress.

Clonidine demonstrates effects on factors attributed to bone marrow dysfunction and anemia in animal studies. As described previously, trauma results in elevated levels of urine norepinephrine in rodents (Bible et al., 2015a; Alamo et al., 2017b; Loftus et al., 2018a). Elevated levels of urine norepinephrine significantly decreased by nearly half among rats that received daily intraperitoneal clonidine injections after CS and LCHS + CS, with *n* = 6–8 per group (Alamo et al., 2017b). In a rodent model of LCHS with or without chronic restraint stress, rodents showed a significant increase in bone marrow expression of HMGB1 and decrease in bone marrow expression of SCF and IL-10 compared to control rats (Loftus et al., 2019). Bone marrow expression of Bcl-xL was not significantly altered after LCHS or LCHS + CS (Loftus et al., 2019). In groups which underwent LCHS or LCHS + CS followed by daily administration of clonidine, bone marrow expression of HMGB1 was significantly decreased by 47%–54% respectively compared to untreated rodents (Loftus et al., 2019). LCHS and LCHS + CS groups treated with clonidine demonstrated a significant increase of 468% and 1,062% in bone marrow SCF; bone marrow Bcl-xL also exhibited a significant increase of 77% in LCHS + CS after clonidine treatment compared to untreated counterparts (Loftus et al., 2019). This study demonstrated how clonidine decreased inhibitory cytokines and growth factors and increased pro-erythropoietic factors, supporting positive effects of this medication on erythropoiesis.

Given these results, another point of interest has been the effects of alpha agonism on HPC mobilization, erythroid progenitor growth, and hemoglobin. In a rodent model of trauma consisting of chronic stress, LCHS, or LCHS and chronic stress with or without daily clonidine administration, groups receiving daily stress who were treated with daily intraperitoneal clonidine demonstrated significantly decreased peripheral HPC by 66% and 84% compared to their untreated counterparts (Alamo et al., 2017b). This study also found significantly decreased growth of CFU-E, BFU-E, and CFU-GEMM in bone marrow-derived cells from all groups subjected to stress and/or trauma, but that such reductions could be prevented with clonidine administration, showing significantly increased levels of CFU-E, BFU-E, and CFU-GEMM when compared to untreated groups (Alamo et al., 2017b). Another study of LCHS with chronic stress also demonstrated an increase in peripheral HPC which was significantly decreased after clonidine administration (Loftus et al., 2020). Factors such as plasma corticosterone, HGF, MMP-9, c-Met, and G-CSF all play roles in HPC mobilization as previously described. Among rats subjected to LCHS with chronic restraint stress, plasma corticosterone levels were significantly elevated compared to controls; these levels were subsequently significantly decreased by 125 ng/ml when administered daily intraperitoneal clonidine compared to untreated counterparts (Loftus et al., 2020). In a rodent model of LC, LCHS, or LCHS and chronic stress with or without clonidine administration, rodents who underwent LC or LCHS + CS showed significantly elevated levels of bone marrow HGF and MMP-9 compared to controls (Loftus et al., 2020). Clonidine administration resulted in significant decline in bone marrow MMP-9 and c-Met compared to untreated rats in LCHS + CS and LC, respectively (Loftus et al., 2020). Levels of bone marrow HGF were not significantly different in rats treated with clonidine compared to untreated (Loftus et al., 2020). In LCHS + CS, plasma G-CSF was significantly elevated and was subsequently decreased by 44% when each group was treated with clonidine (Alamo et al., 2017b). Therefore, clonidine decreases HPC mobilization and inhibits pro-mobilizing factors such as plasma G-CSF, bone marrow c-Met and bone marrow MMP-9. Finally, rodent studies using lung contusion and hemorrhagic shock with or without daily restraint stress have identified significant reductions of 1–4 g/dl in hemoglobin compared to uninjured control rats, which was not observed with clonidine administration (Alamo et al., 2017b; Loftus et al., 2020). Rats treated with clonidine demonstrated mean hemoglobin levels which were 1–2 g/dl greater than untreated rats (Alamo et al., 2017b; Loftus et al., 2020). Alpha agonism not only contributes to suppression of the cytokines that affect erythropoiesis but also decreases mobilization of HPC and improves hemoglobin.

Few human studies have investigated the effects of clonidine on bone marrow dysfunction and anemia in trauma patients. In a retrospective cohort analysis of almost 300 human trauma patients requiring admission to a SICU, those who received clonidine and/or a non-selective beta blocker for at least 25% of their stay demonstrated a significant improvement in hemoglobin at time of discharge compared to trauma patients who had not received those medications, an absolute difference of almost 0.5 g/dl (Loftus et al., 2018c). However, patients in this study were pooled into one treatment group and not separated by medication, and only 7% of this group received clonidine alone (Loftus et al., 2018c). Therefore, the specific effect of clonidine on minimizing anemia cannot be determined from the findings of Loftus et al. (2018c). However, clonidine has the potential to play a role in the promotion of erythropoiesis in trauma patients.

### 3.2 Beta Blockade Following Trauma

#### 3.2.1 Beta-1 Blockade

Beta-1 receptors are the predominant adrenoreceptors found in the human heart (Brodde, 2008). Drugs that bind to and inhibit β-1 receptors are used in the treatment of hypertension, coronary artery disease and angina (Brodde, 2008). Atenolol selectively inhibits β-1 receptors in the heart, resulting in decreased blood pressure, heart rate and cardiac output (Heel et al., 1979). Unfortunately, and perhaps due to its cardioselectivity, β-1 blockade does not appear to have significant effects on plasma G-CSF, bone marrow erythroid progenitor growth, bone marrow cellularity or mobilization of HPC in rodent trauma models (Beiermeister et al., 2010; Pasupuleti et al., 2012; Pasupuleti et al., 2014; Miller et al., 2020). Only one rodent model of LCHS + CS with post-injury atenolol administration demonstrated significantly decreased plasma G-CSF levels by 89% compared to the untreated trauma group (Miller et al., 2020). Other studies did not show changes in plasma G-CSF with atenolol administration to injured rats (Pasupuleti et al., 2012; Pasupuleti et al., 2014). In rodent trauma studies of LCHS with and without chronic stress, treatment with intraperitoneal atenolol after resuscitation failed to improve the increased HPC mobilization, decreased bone marrow cellularity, and restricted erythroid progenitor growth (CFU-E, BFU-E, CFU-GEMM) exhibited 3 hours after injury (Pasupuleti et al., 2012; Pasupuleti et al., 2014; Miller et al., 2020). In one study, rats pretreated with atenolol for 3 days prior to lung contusion demonstrated similar derangements in HPC mobilization and erythroid progenitor growth suppression as their untreated counterparts 3 hours after injury (Beiermeister et al., 2010). This suggests that β-1 receptors do not have a substantial impact of erythroid progenitor growth recovery in the bone marrow or reduction in HPC egress from the bone marrow to sites of injury.

#### 3.2.2 Beta-2 Blockade

Beta-2 receptors are located in human bronchial smooth muscle cells, vascular smooth muscle cells and cardiac myocytes, albeit at a lower expression density compared to beta-1 receptors (Brodde, 2008; Perez, 2021). While selective β-2 antagonists are not in clinical use in humans, a beta-2 blocking agent called butoxamine has been shown to decrease blood pressure and heart rate and increase airway resistance in animal studies (Letts et al., 1983; Beiermeister et al., 2010).

The effects of selective blockade of β-2 receptors on erythropoiesis have been studied in rodent models of both burn and trauma with promising results. Such studies have assessed bone marrow cellularity, erythroid progenitor growth, erythroblasts, multi-potent progenitor MafB expression, HPC mobilization, plasma G-CSF, and hemoglobin (Beiermeister et al., 2010; Pasupuleti et al., 2014; Hasan et al., 2017b; Miller et al., 2020). In a study of rats subjected to LCHS with or without chronic stress sacrificed at 3 h or 7 days, post-resuscitation intraperitoneal butoxamine administration significantly increased overall bone marrow cellularity and erythroid progenitor growth compared to rats who were injured but did not receive the drug (Pasupuleti et al., 2012; Pasupuleti et al., 2014; Miller et al., 2020). Treated groups had significantly decreased plasma G-CSF by 95% and HPC mobilization by 54% compared to untreated groups (Miller et al., 2020). Pasupuleti et al. (2014) also demonstrated a significant decrease in HPC mobilization after LCHS and administration of butoxamine. Hemoglobin levels in treated rats who underwent LCHS + CS were 12% higher than their untreated counterparts (Miller et al., 2020). Pre-treatment with butoxamine for 3 days prior to injury eliminated injury-induced changes in HPC egress or erythroid progenitor growth in the bone marrow (Beiermeister et al., 2010). Similarly, mice subjected to scald burn and subsequently administered daily butoxamine for 6 days before sacrifice demonstrated increased early and late erythroblasts, overall erythroid cell count and hemoglobin compared to mice who sustained burn injury alone without treatment (Hasan et al., 2017b). In this murine model, multipotent progenitors exhibited reduced expression of the transcription factor MafB after administration of butoxamine compared to untreated mice (Hasan et al., 2017b). MafB induces monocytic differentiation which diverts multipotent progenitor cells from erythropoiesis (Howell et al., 2012). Thus, β-2 blockade appears to play a role in the decreased mobilization of HPC and also promotes erythropoiesis, proving to have potential clinical use in trauma patients to prevent anemia.

#### 3.2.3 Beta-3 Blockade

Our understanding of the function of beta-3 receptors in specific tissues remains a topic of ongoing investigation. In humans, they are involved in lipolysis and thermogenesis in adipose tissue; they are also present in the myocardium and tissues of the urinary system and central nervous system (Schena and Caplan, 2019). The inotropic and/or chronotropic effects of beta-3 adrenoceptor stimulation are not well understood—rodent studies have shown either minimal or even negative inotropic effects, though this is an ongoing topic of study (Arioglu-Inan et al., 2019). Until recently, selective beta-3 antagonists such as SR59230A and L748337 were only approved for research purposes; Mirabegron (YM178) is the only FDA-approved selective beta-3 antagonist currently available for prescription, indicated for overactive bladder syndrome (Schena and Caplan, 2019).

Similar to other selective adrenergic receptor blocking agents, data thus far on the link between beta-3 receptor blockade and erythropoiesis has been limited to rodent studies. When administered SR59230A after resuscitation, rats subjected to LCHS with or without chronic restraint stress showed no injury-induced reductions in bone marrow cellularity or erythroid progenitor growth (Pasupuleti et al., 2014; Miller et al., 2020). SR59230A was associated with decreased plasma G-CSF and HPC mobilization compared to rats who had not received the agent (Pasupuleti et al., 2014; Miller et al., 2020). Finally, the model of LCHS with chronic stress demonstrated that rodents who received SR59230A had a significantly higher hemoglobin, a 10% increase, compared to trauma rodents (Miller et al., 2020). In another study, pre-treatment of rodents with SR59230A for 3 days prior to lung contusion eliminated suppression of erythroid progenitor growth (including CFU-E, BFU-E, and CFU-GEMM) (Beiermeister et al., 2010). Pretreatment also effectively eliminated the postinjury rise in HPC mobilization out of the bone marrow (Beiermeister et al., 2010). Mice administered the beta-3 antagonist SR59230A for 6 days after scald burn injury demonstrated higher levels of early erythroblasts, reticulocytes, overall erythroid cell count, and hemoglobin compared to untreated rodents (Hasan et al., 2017b). These findings support a role for beta-3 receptor blockade in enhancing erythropoiesis after an insult, although human trials have not yet been completed.

#### 3.2.4 Non-selective Beta Blockade

While selective beta-2 and beta-3 antagonism have shown some promise in improving erythropoietic activity after acute injury, the best-studied and most promising of all has been non-selective beta receptor blockade. Drugs such as nadolol and propranolol are used in the treatment of hypertension, arrhythmias, angina, and other cardiovascular diseases (Rosendorff, 1993; Prichard et al., 2001). Propranolol has been found to improve cerebral perfusion and decrease cerebral hypoxia in traumatic brain injury, restore axonal function after spinal cord injury, and reduce post-injury hyperglycemia (Loftus et al., 2016). It has been linked to faster wound healing, reduced surface area required for skin grafting, reduced blood loss during grafting procedures, and decreased overall hospital length of stay after burn injuries (Mohammadi et al., 2009; Ali et al., 2015). Propranolol, when dosage is titrated to heart rate, reduces muscle-protein catabolism in pediatric burn patients (Herndon et al., 2001). Due to propranolol’s particular appeal as a well-tolerated and clinically useful medication with a variety of applications for critically ill and injured patients, it has been well-studied in humans and animals as a potential agent to prevent post-injury anemia.

Non-selective beta antagonism reduces the systemic inflammatory response after trauma. A rodent trauma model of LCHS with chronic stress found an increase in bone marrow IL-1β after injury which did not occur in rats who received intraperitoneal propranolol (Apple et al., 2020b). Rat models of LCHS with chronic restraint stress exhibit increased expression of bone marrow nitric oxide synthase and TNFα and elevated plasma CRP (Apple et al., 2020b). When administered 10 minutes post-injury and daily thereafter, propranolol led to significantly decreased levels of bone marrow TNFα and nitric oxide synthase, and plasma CRP compared to untreated animals (Apple et al., 2020b). Finally, non-selective beta blockade with propranolol also significantly upregulated bone marrow microRNAs linked to suppression of inflammatory cytokines in rats (Apple et al., 2020b).

Propranolol has been studied to return dysregulated iron pathways to homeostasis. Rats subjected to LCHS followed by chronic restraint stress demonstrated decreased circulating levels of the iron-binding protein transferrin, as well as its most widespread receptor transferrin receptor 1 (TFR-1); it decreased liver ferroportin expression, bone marrow transferrin expression, and paradoxically plasma hepcidin in LCHS with and without chronic stress (Alamo et al., 2017a; Sangkhae and Nemeth, 2017). Administration of propranolol resulted in significantly elevated levels of liver ferroportin, bone marrow transferrin, and bone marrow TFR-1 compared to untreated counterparts, although it paradoxically resulted in elevated hepcidin levels in both LCHS and LCHS + CS compared to untreated counterparts (Alamo et al., 2017a). These findings suggest that propranolol plays a role in maintenance of iron homeostasis after trauma.

Many animal studies have been performed to evaluate the effects of non-selective beta blockade on HPC mobilization. Rodent studies of trauma demonstrate HPC mobilization to the peripheral blood, measured by flow cytometry of CD71+/c-kit + cells, and elevated plasma G-CSF in rats sacrificed at 3 hours and 24 h (Baranski et al., 2011; Baranski et al., 2013). Administration of propranolol decreased plasma G-CSF by 50% and HPC mobilization significantly compared to untreated groups (Baranski et al., 2011; Baranski et al., 2013). Similar findings of decreased plasma and bone marrow G-CSF and HPC mobilization were also observed in models consisting of injury followed by 7 days of daily propranolol administration (Bible et al., 2015b; Loftus et al., 2020; Doobay et al., 2021). One study investigated the importance of timing of propranolol administration to rodents after injury and how this affected HPC mobilization and plasma G-CSF (Baranski et al., 2013). When propranolol was administered immediately or 1 hour after resuscitation with autologous blood transfusion, HPC mobilization and plasma G-CSF were significantly decreased compared to untreated counterparts (Baranski et al., 2013). One study has investigated pre-treatment of rodents with propranolol for 3 days prior to lung contusion alone; this showed decreased HPC mobilization as well (Beiermeister et al., 2010). These studies provide evidence that when either administered prior to or after injury, propranolol inhibits HPC mobilization and decreases plasma G-CSF levels.

Various factors play roles in HPC mobilization as previously discussed. Trauma rodent models demonstrate elevation of bone marrow HMGB1, HGF, and MMP-9 along with plasma corticosterone (Loftus et al., 2019; Loftus et al., 2020). After receiving intraperitoneal propranolol for 6 days after injury, levels of most of these factors were decreased, with the exception of bone marrow HGF which showed no change (Loftus et al., 2019; Loftus et al., 2020). Another rodent model of LCHS showed increased plasma MMP-9 levels at 3 hour which were significantly decreased in rodents treated with propranolol post-injury (Baranski et al., 2011). Finally, a 7 day rodent trauma model showed that propranolol significantly decreased bone marrow expression of HMGB1, MMP-2, MMP-9, and neutrophil elastase and also plasma neutrophil elastase compared to rodents who did not receive the drug (Doobay et al., 2021). This study also demonstrated decreased bone marrow expression of TIMP-1 and TIMP-2, possibly in response to suppressed bone marrow expression of matrix metalloproteinases (Doobay et al., 2021). Regarding factors which promote retention of HPC in bone marrow, VLA-4 (LCHS + CS) SDF-1 (LC) and CXCR4 (LCHS + CS) were paradoxically reduced by propranolol and had no effects on VCAM-1 (Doobay et al., 2021). This suggests that expression of these molecules which play a role in retention of HPC in bone marrow may not be directly affected by adrenergic modulation.

Propranolol also subsequently affects bone marrow cellularity, bone marrow erythroid progenitor growth and hemoglobin. Rodent trauma models of various combinations of LC and/or HS with or without chronic stress result in reduction of bone marrow cellularity; this suppression is not observed with administration of propranolol (Baranski et al., 2011; Baranski et al., 2013; Alamo et al., 2017a). These models have also consistently demonstrated suppression of bone marrow CFU-GEMM, BFU-E, and CFU-E (Beiermeister et al., 2010; Elhassan et al., 2011; Mohr et al., 2011; Baranski et al., 2013; Alamo et al., 2017a). Rodents pre-treated with propranolol demonstrated significantly higher levels of bone marrow erythroid progenitor cells compared to untreated groups (Beiermeister et al., 2010; Elhassan et al., 2011). Rodents administered post-injury propranolol also prevented suppression of erythroid progenitor growth when administered immediately or 1 hour after resuscitation (Baranski et al., 2013). In rodent trauma models with administration of daily propranolol for days before sacrifice, propranolol also prevented suppression of erythroid progenitor growth seen in untreated rodents (Elhassan et al., 2011; Mohr et al., 2011; Alamo et al., 2017a). Murine models have also demonstrated that when non-selective beta blockade was administered daily post burn for a 7-day time period, erythroid cells increased (Hasan et al., 2017b). There was decreased expression of MafB in burn mice administered propranolol, preventing monocytic differentiation of multipotent HPC (Hasan et al., 2017a; Hasan et al., 2017b). Finally, rats who underwent LCHS and LCHS + CS who were administered propranolol had improved hemoglobin levels, with increases of up to 3 g/dl, versus untreated groups (Mohr et al., 2011; Alamo et al., 2017a; Loftus et al., 2020). Thus, non-selective beta blockade not only supports bone marrow cellularity and erythroid progenitor growth but can also lead to improvement of anemia.

Investigation has also been conducted on factors which affect erythropoiesis such as SCF, Bcl-xL, and erythropoietin. Rodent trauma models of LCHS and with and without chronic stress show that bone marrow expression of both SCF and Bcl-xL are significantly increased in rodents receiving propranolol after injury (Loftus et al., 2019). In addition, there is a reflexive increase in plasma erythropoietin after traumatic injury in rodents who undergo LCHS which is suppressed with administration of propranolol (Alamo et al., 2017a). As such, propranolol mediates the increase of some pro-erythropoietic factors such as bone marrow SCF and Bcl-xL; it also prevents the post-injury surge in plasma erythropoietin. Although seemingly paradoxical for propranolol to prevent a surge in plasma erythropoietin, it could be hypothesized that the benefits in erythroid growth and hemoglobin seen with non-selective beta blockade prevent the burst of erythropoietin seen after hemorrhage.

Few human studies have been performed in the effects of non-selective beta blockade on erythropoiesis after injury. In a study of burn patients who received propranolol during their hospital stay, evaluation of serial blood samples demonstrated increased numbers of erythroid progenitors and decreased MafB expression in multipotent progenitors (Hasan et al., 2017a). In a separate study, burn patients who received propranolol as part of their treatment were found to have greater numbers of erythroblasts and alpha hemoglobin stabilizing protein, which is associated with late stage maturation of erythroblasts, compared to patients who had not received propranolol (Walczak et al., 2021). Burn patients who received propranolol also were found to have significantly fewer blood transfusion requirements during their hospitalization (Walczak et al., 2021). A four-year retrospective cohort analysis of trauma patients administered non-selective beta blockade and/or clonidine demonstrated significantly higher hemoglobin at time of discharge compared to trauma patients who did not receive either medication; although notably this study did not separate groups into the medication received, the majority (81%) of patients had exclusively received a non-selective beta blocker (Loftus et al., 2018c). In a small randomized trial of severely injured trauma patients administered propranolol titrated to a 10%–20% decrease in heart rate, serial blood samples demonstrated significantly lower peripheral BFU-E and CFU-E compared to trauma patients who did not receive propranolol (Bible et al., 2014). Despite a 1 g/dl improvement in discharge hemoglobin in trauma patients treated with propranolol compared to untreated patients, this finding was not statistically significant; in addition, there was no difference in blood transfusion requirement between the two groups (Bible et al., 2014). However, these results do illustrate the significance of propranolol administration to improve hemoglobin in trauma patients.

## 4 Conclusion and Future Directions

Adrenergic modulation in trauma patients promotes erythropoiesis through multiple mediators with alpha agonism and beta antagonism. The most promising results from animal and human studies of such drugs to promote erythropoiesis are from alpha agonism and non-selective beta blockade of beta-1 and beta-2 receptors. Alpha agonism in animals increases cytokines promoting erythropoiesis (SCF, Bcl-xL) and decreases inhibitory cytokines (HMGB1), decreases the peripheral mobilization of HPC and increases bone marrow cellularity, erythroid progenitor growth, and hemoglobin, though human studies are needed to demonstrate similar results in trauma patients (Alamo et al., 2017b; Loftus et al., 2020). Non-selective beta blockade with propranolol dominates this area of study, with promising results in preventing proinflammatory cytokine release, improving iron homeostasis, reducing pro-mobilization factors, and improving hemoglobin in rodents as shown in Table 1, as well as a few limited but promising human studies. Selective beta blockade with β-1, β-2 and β-3 antagonists have varying effects on erythropoiesis and related factors. Beta-1 blockade with atenolol after injury in rodents does not show significant changes in bone marrow cellularity, bone marrow erythroid progenitor growth, HPC mobilization, or plasma G-CSF compared to untreated rodents (Pasupuleti et al., 2012; Pasupuleti et al., 2014; Miller et al., 2020). Beta-2 and beta-3 adrenergic receptors appear to be involved in HPC mobilization mediated by various factors, and selective β-2 and β-3 antagonism shows some promise in improving bone marrow cellularity, erythroid progenitor growth, and perhaps hemoglobin (Pasupuleti et al., 2012; Pasupuleti et al., 2014; Miller et al., 2020). Unfortunately, these two selective beta antagonist medications are not in clinical use. The results from these studies warrant further investigation in human research of erythropoiesis after trauma.

There still is a need for further studies of the use of adrenergic modulation in specific trauma types and different age groups. The human studies of adrenergic modulation of erythropoiesis presented have been performed in both blunt trauma and burn patients (Bible et al., 2014; Hasan et al., 2017a; Loftus et al., 2018c; Walczak et al., 2021). However, no studies to date have investigated the use of alpha agonists or beta antagonists and effects on erythropoiesis in patients subject to penetrating trauma. In addition, the human studies which have been performed are of patients in a wide range of ages and did not examine differences between age groups. Study of the use of these agents in penetrating trauma and comparison between age groups should be performed.

Despite the number of rodent studies which have been performed to evaluate various effects of adrenergic modulation on erythropoiesis, no studies to date have been performed in female animals. Human studies on erythropoiesis after trauma have not assessed differences between males and females in response to adrenergic modulation after trauma, despite different physiologic reactions to these medications between sexes. In a human study, esmolol, a beta-1 antagonist, significantly decreased the mean arterial pressure of males but not females in the setting of adrenergic stimulation (Coulson and Cockcroft, 2011). In addition, men have shown increased sensitivity to propranolol and exhibited a more pronounced drop in blood pressure compared to women after exercise (Samora et al., 2019). These studies suggest a role for sex hormones in the physiologic response to these adrenergic medications, thus warranting further study of differences in response to adrenergic modulation in the setting of erythropoiesis after trauma between sexes.

## Glossary

Bcl-xL: B-cell lymphoma-extra large
BFU-E: Erythroid burst-forming units
CFU-E: Erythroid colony-forming unit
CFU-GEMM: Multipotential colony-forming unit
c-Met: Tyrosine-protein kinase Met
CMP: Common myeloid progenitor
CRP: C-reactive protein
CS: Chronic restraint stress
CXCL12: C-X-C motif chemokine ligand 12
CXCR4: C-X-C chemokine receptor type 4
EBI: Erythroblastic Island
G-CSF: Granulocyte-colony stimulating factor
HGF: Hepatocyte growth factor
HMGB1: High-mobility group box-1
HPC: Hematopoietic progenitor cell
HS: Hemorrhagic shock
HSC: Hematopoietic stem cell
IL-10: Interleukin-10
IL-1α: Interleukin-1 alpha
IL-1β: Interleukin-1 beta
IL-2: Interleukin-2
IL-6: Interleukin-6
LC + CS: Lung contusion with chronic restraint
LC: Lung contusion
LCHS + CS: Lung contusion with hemorrhagic shock and chronic restraint stress
LCHS: Lung contusion with hemorrhagic shock
MEP: Megakaryocyte erythroid progenitor
MMP-2: Matrix-metalloproteinase-2
MMP-9: Matrix-metealloproteinase-9
NO: Nitric oxide
SCF: Stem cell factor
SDF-1: Stromal-cell derived factor-1
SICU: Surgical intensive care unit
TGF-β: Transforming growth factor beta
TIMP-1: Tissue inhibitor of metalloproteinase-1
TIMP-2: Tissue inhibitor of metalloproteinase-2
TNF-α: Tumor necrosis factor alpha
TRF-1: Transferrin receptor-1
VCAM-1: Vascular cell adhesion molecule-1
VLA-4: Very late antigen-4
